# Diet and Lifestyle Factors Associated with Gastrointestinal Symptoms in Spanish Adults: Cross-Sectional Analysis of the 2023 Spanish National Health Survey

**DOI:** 10.3390/nu18020299

**Published:** 2026-01-17

**Authors:** Ángel López-Fernández-Roldán, Víctor Serrano-Fernández, José Alberto Laredo-Aguilera, Esperanza Barroso-Corroto, Laura Pilar De Paz-Montón, Juan Manuel Carmona-Torres

**Affiliations:** 1 Facultad de Fisioterapia y Enfermería, Universidad de Castilla-La Mancha, 45071 Toledo, Spain; angel.lopezfernandez@uclm.es (Á.L.-F.-R.); josealberto.laredo@uclm.es (J.A.L.-A.); esperanza.barroso@uclm.es (E.B.-C.); laurapilar.paz@uclm.es (L.P.D.P.-M.); juanmanuel.carmona@uclm.es (J.M.C.-T.); 2 Grupo de Investigación Multidisciplinar en Cuidados (IMCU), Universidad de Castilla-La Mancha, 45071 Toledo, Spain; 3 Instituto de Investigación Sanitaria de Castilla-La Mancha (IDISCAM), 45004 Toledo, Spain

**Keywords:** lifestyle, dietary intake, gastrointestinal symptoms, prevalence, cross-sectional study

## Abstract

Background/Objectives: Digestive problems are common in the general population and may be influenced by lifestyle, emotional status and diet. This study aimed to estimate the prevalence of digestive problems in Spanish adults and examined associated factors. Methods: Descriptive cross-sectional analysis of anonymized adult microdata from the 2023 Spanish Health Survey was performed. Data were collected using a mixed-mode design (self-administered web questionnaire with interviewer-administered follow-up). Digestive problems were recoded by combining gastric ulcer, constipation, and prescribed use of laxatives, astringent drugs, and stomach medication. Therefore, digestive problems are primarily defined as the presence of gastric ulcers, diarrhea, and/or constipation. Variables included sociodemographic, Body Mass Index (BMI), smoking, alcohol intake, physical activity, Personal Health Questionnaire Depression Scale (PHQ-8), World Health Organization Well Being Index (WHO-5), and macronutrient intake estimated from a Food-Frequency Questionnaire using the Spanish Food Composition Database (BEDCA). Group comparisons and multivariable logistic regression were conducted (95% CI; significance level set at *p* < 0.05). Results: Of 34,148 participants, 13,518 provided information on digestive problems; among these respondents, 3860 (28.6%) reported having digestive issues. Prevalence ranged from 5.2% to 36.5% among national territories. Higher odds (OR) of digestive problems were associated with age (OR 1.026, 95% CI 1.023–1.029), female sex (OR 1.168, 1.070–1.276), non-smoking (OR 1.240, 1.005–1.531) and ex-smoking (OR 1.447, 1.272–1.647) compared to current smokers, higher PHQ-8 scores (OR 1.040, 1.029–1.051), greater protein intake (OR 1.016, 1.009–1.023), consumption of sweet pastries (OR 1.058, 1.039–1.077), and dairy products (OR 1.027, 1.002–1.053); in contrast, lower odds were associated with higher WHO-5 scores (OR 0.985, 0.982–0.987), total fiber intake (OR 0.968, 0.949–0.987), and legume consumption (OR 0.894, 0.856–0.933). Conclusions: Digestive problems show considerable variability in prevalence among survey-based Spanish sample. Digestive problems were associated with older age, female sex, depressive symptoms, high-protein intake, and higher consumption of sweet pastries and dairy products, whereas higher well-being scores, higher fiber intake and legume consumption were associated with lower odds of digestive problems.

## 1. Introduction

Gastrointestinal symptoms and disorders are common in the general population and are associated with increased healthcare resource utilization and reductions in patients’ quality of life [1,2]. Their impact on quality of life is primarily due to physical, psychological, and social limitations, as these conditions directly affect sleep, perceived fatigue, nutrient deficiencies, stress, and social avoidance [3,4,5,6].

Some of the most frequent digestive problems include diarrhea, constipation, nausea, vomiting, and gastric ulcers [7,8,9]. The incidence of various gastrointestinal disorders depends on geographic region and the specific type of condition [1,8,10,11,12]. Specifically, in areas with low and medium sociodemographic indices—such as South Asia, Africa, and several regions of the Middle East—infectious diarrhea predominates, closely linked to poverty, sanitation, and limited access to healthcare services [11,12]. Conversely, in regions with a high sociodemographic index, such as United States and European Countries, the most prevalent gastrointestinal conditions are chronic in nature, including colorectal cancer, Inflammatory Bowel Disease (IBD), Irritable Bowel Syndrome (IBS), and functional gastrointestinal disorders such as dyspepsia [1,10].

In Spain, the most frequent digestive disorders include those related to the gut–brain interaction—functional constipation, dyspepsia, and gastroesophageal reflux—as 43.6% of Spanish adults meet diagnostic criteria for at least one of these conditions [13]. Specifically, constipation may occur in 12.8% of the population, dyspepsia in 7.1%, and reflux in 31.6% of Spanish adults [13]. On the other hand, diarrhea is generally attributed to infectious etiologies, with a prevalence of 3.4% due to enteroaggregative Escherichia coli [14], while other conditions such as gastric ulcer are commonly associated with Helicobacter pylori infection, reaching a prevalence of up to 4% [15].

Regarding risk factors for digestive diseases, habits such as smoking are linked to an increased risk of gastric ulcer, the development of related complications [16,17], and functional symptoms such as constipation [18]. However, in conditions like IBD, its effect is controversial, as smoking acts as a risk factor for Crohn’s disease but appears to be protective against ulcerative colitis [19]. Additionally, other harmful habits such as alcohol consumption have been associated with problems including diarrhea, malabsorption, nutritional deficiencies [20], and gastric ulcers [17].

Moderate-intensity physical activity (PA) has been associated with improved gastrointestinal function and lower rates of constipation and abdominal pain [21,22]. This effect is attributed to its role in regulating intestinal motility through modulation of the enteric nervous system, reduction in systemic inflammation, and microbiota modulation, promoting bacterial diversity [21,23].

Furthermore, obesity is a factor linked to an increased risk of chronic diarrhea [24], as excess body fat enhances the production of inflammatory mediators such as adipokines, TNF- α, IL-1β or IL-6, which contribute to low-grade chronic inflammation [25,26].

Regarding emotional and digestive problems, their relationship is bidirectional [27]. Conditions such as gastric ulcer, constipation, and diarrhea can be influenced by emotional states through the gut–brain–microbiota axis, mediated by communication between the enteric nervous system and the central nervous system [28,29]. This interaction is primarily driven by neurotransmitters such as serotonin, dopamine, and gamma-aminobutyric acid, as well as microbial metabolites like short-chain fatty acids [29,30,31].

Diet plays a fundamental role in digestive health, as the intake of certain nutrients such as fiber helps regulate intestinal motility [32]. Moreover, high-fiber diets promote microbial diversity and the production of short-chain fatty acids that exert anti-inflammatory effects [33,34]. However, excessive consumption of saturated fats, sugar, refined carbohydrates, and animal proteins may pose a risk to digestive health, as these dietary patterns increase intestinal permeability, induce dysbiosis, and elevate the production of inflammatory mediators, which are associated with symptoms such as diarrhea and abdominal pain [35,36,37].

As previously discussed, several factors are associated with the onset of digestive problems [13,14,15]. Understanding these factors is essential from a public health perspective to enable targeted interventions and reduce the prevalence of digestive disorders. Regarding prevalence, to the best of our knowledge, no studies have examined this concept in conjunction with health-related behaviors in a nationally representative sample.

Therefore, the objectives of this study were to estimate the prevalence of digestive problems among adults in Spain and to assess their association with factors such as smoking, alcohol consumption, physical activity, obesity, emotional well-being, and macronutrient intake.

## 2. Materials and Methods

### 2.1. Design and Participants

A descriptive cross-sectional study was conducted. The data source was individuals who participated in the 2023 Spanish Health Survey (EsdE 2023) [38]. This survey consists of interviews carried out by the National Statistics Institute in collaboration with the Ministry of Health.

The ESdE 2023 uses a three-stage probabilistic sampling design, with stratification of census sections in the first stage and family dwellings in the second stage. Finally, in the last stage, individuals are randomly selected within households using the Kish method, which assigns equal probability to all adults. Participants are first invited to complete the questionnaire online and households that do not respond are subsequently followed up with a computer-assisted personal interview.

### 2.2. Variables

Sociodemographic variables collected included age, sex, educational level, employment status, and social class.

The dependent variable of interest was the presence of digestive problems. This variable was recategorized from other variables in the database by combining the presence of gastric ulcer, constipation, medical prescription of laxatives, astringent drugs, and stomach medication, as these were the aspects assessed by the EsdE 2023.

Independent variables included those related to participants’ health status and behaviors. Specifically, Body Mass Index (BMI), weight status, smoking habits, number of cigarettes smoked per day, number of alcoholic drinks consumed per week, physical activity level, depression, mental well-being, and dietary nutrient intake were analyzed.

### 2.3. Instruments

The tools used for data assessment varied according to the type of variable analyzed. For social class, the National Classification of Occupations 2011 was applied which distinguishes six social classes based on the level of occupational responsibility [39].

PA level was measured using the International Physical Activity Questionnaire (IPAQ) [40]. This instrument includes eight items that assess the type and duration of PA and classifies it as low, moderate, or vigorous. Moderate PA is defined as meeting any of the following criteria: (1) three or more days of vigorous activity for at least 20 min per day; (2) five or more days of vigorous activity for at least 30 min per day; or (3) five or more days of any combination of walking, moderate, or vigorous activity achieving a total of at least 600 METs (the unit of measurement used by the test). Vigorous PA is defined as (1) performing this type of activity at least three days per week with a minimum total of 1500 METs, or (2) seven days of any combination of walking, moderate, and/or vigorous activity achieving at least 3000 METs. Finally, low PA or inactivity is classified when none of the above criteria are met.

Emotional assessment was performed using the Personal Health Questionnaire Depression Scale (PHQ-8) [41] and the World Health Organization Well-Being Index (WHO-5) [42]. The PHQ-8 consists of eight items scored from 0 (never) to 3 (every day). Scores above 10 indicate depression, and scores above 20 indicate severe depression [41]. The WHO-5 index comprises five items scored from 0 (never) to 5 (all the time). The final score is multiplied by four, with scores below 50 indicating low well-being and scores above 50 indicating adequate well-being [42].

Nutritional assessment was based on a Food Frequency Questionnaire (FFQ) developed by the National Statistics Institute and the Ministry of Health [38]. For macronutrient calculation, reported food consumption frequencies were combined with the average macronutrient content per edible portion of each food group, following procedures similar to previous studies [43,44]. Macronutrient estimates were obtained using the Spanish Food Composition Database (BEDCA) [45], an open access national database that compiles food composition values from multiple sources and is coordinated within Spain’s institutional framework for food safety and nutrition. Energy and macronutrient intake estimates were calculated in SPSS (version 29) by assigning BEDCA-derived nutrient values to each FFQ food item and aggregating the corresponding totals across all items.

Additionally, we estimated consumption frequencies for all food groups assessed in the survey, allowing us to report weekly intake for each specific group.

### 2.4. Procedure

The anonymized microdata file was downloaded from the National Statistics Institute website, which is publicly accessible and does not require special permissions for use. After downloading, the database was cleaned and processed using IBM SPSS Statistics v29 under a license from the University of Castilla-La Mancha.

### 2.5. Statistical Analysis

Data analysis was performed using IBM SPSS Statistics v29. Qualitative variables were expressed as frequencies (n) and percentages (%). Quantitative variables were expressed as arithmetic mean (m) and standard deviation (SD).

Categorical variables were compared using the Chi-square test. Normality was evaluated using the Shapiro–Wilk test, histograms, and Q-Q plots. Additionally, bivariate correlations between continuous quantitative variables were calculated using Pearson’s correlation coefficients.

Variables found to be statistically significant were included in a logistic regression model to identify odds ratios (OR) representing the risk of presenting digestive problems. Along with the regression model, Nagelkerke’s R^2^ was reported to indicate model variability.

Dietary variables were analyzed as absolute intakes and were not adjusted for total energy intake. Effect sizes for nutritional differences were calculated using Cohen’s d, along with their corresponding 95% confidence intervals (CI).

All hypothesis tests were two-tailed, and results were considered statistically significant at *p* < 0.05 within a 95% CI.

Missing data were handled using a complete-case approach. Analyses were restricted to participants with non-missing information on digestive problems, and for each statistical test, only individuals with complete data for the variables involved were included.

### 2.6. Ethical Considerations

The data collected in EsdE 2023 are publicly available for download in anonymized form through the National Statistics Institute website. As these data are accessible to the general population, their analysis does not require approval from an ethics committee in accordance with current Spanish legislation.

## 3. Results

### 3.1. Sample Characteristics

The database included a total of 34,148 participants. Specifically, the analysis was on responses from 13,518 participants who indicated whether they experienced digestive problems. The remaining participants were excluded because they did not answer any questions related to digestive issues.

Among the 13,518 participants, 41.5% were male and 58.5% were female, with a mean age of 60.22 ± 18.10 years. Regarding digestive problems, these were reported by 3860 individuals (28.6%), while the remaining 9658 (71.4%) stated they did not suffer from such conditions. Most participants had completed secondary education (31.5%), were married (72.2%), received some type of pension (32.2%), and belonged to social class 5, corresponding to skilled workers in the primary sector (31.3%). Table 1 provides detailed sociodemographic characteristics of participants according to digestive problem status (yes, no, and non-specified).

### 3.2. Prevalence of Digestive Problems

**Figure 1:**
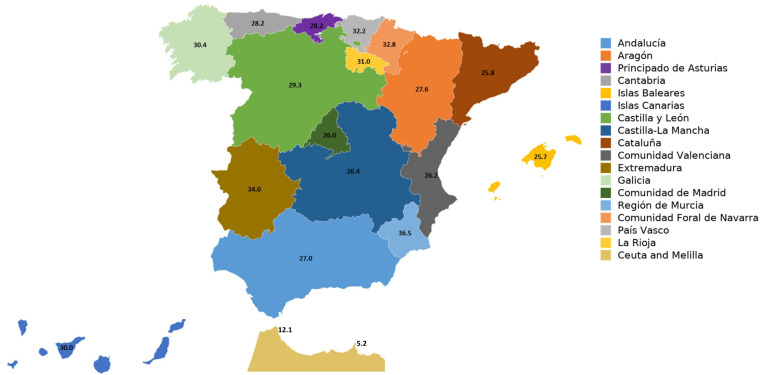
Prevalence (%) of digestive problems by autonomous city/community in Spain.

In the overall sample, digestive problems showed a general prevalence of 28.6%. The communities with the highest prevalence rates were Region de Murcia (36.5%), Extremadura (34.0%), and Comunidad Foral de Navarra (32.8%), while the lowest rates were reported in Melilla (5.2%), Ceuta (12.1%), Islas Baleares (25.7%), and Cataluña (25.8%). Figure 1 illustrates the distribution of digestive problems across Spanish regions.

### 3.3. Differences in Health and Nutritional Habits

Significant differences were found (*p* < 0.001) regarding BMI, with mean values of 26.96 ± 4.82 kg/m^2^ in the group with digestive problems compared to 26.32 ± 4.44 kg/m^2^ in the healthy group, mean difference 0.64 kg/m^2^ (95% CI, 0.46 to 0.82). Weight status also showed significant differences between groups, with higher obesity rates in the group with digestive problems (22.4% vs. 17.9%; *p* <0.001).

Regarding tobacco use, the percentage of smokers was higher among healthy individuals (19.5% vs. 14.1%, *p* < 0.001), whereas the proportion of former smokers was greater in the group with digestive disorders (32.0% vs. 25.6%; *p* <0.001). Daily cigarette consumption was higher among participants without digestive problems, with a mean of 14.06 ± 13.44 cigarettes (median 10, interquartile rank 7–20), compared to 13.77 ± 13.55 cigarettes (median 10, interquartile rank 6–20) in the group with digestive issues; however, this comparison was not statistically significant (*p* = 0.977), and the medians were identical: 10 cigarettes/day (interquartile rank 7–20) among participants without digestive problems vs. 10 cigarettes/day (interquartile rank 6–20) among those with digestive problems. In contrast, the number of alcoholic drinks consumed per week did not show statistically significant differences (*p* = 0.716).

PA levels were higher in the group without digestive problems, with 19.8% and 21% engaging in moderate and vigorous PA, respectively, compared to 14.8% and 16.4% in the group with digestive problems (*p* < 0.001).

Regarding emotional health, the group with digestive problems reported higher PHQ-8 scores compared to the healthy group (7.10 ± 5.98 vs. 4.70 ± 4.61; *p* < 0.001), and the prevalence of depression and severe depression was also greater (22.4% vs. 12.3% vs. 5.6% vs. 1.6%; *p* < 0.001). Similarly, WHO-5 well-being scores were lower in the group with digestive problems (59.47 ± 25.11 vs. 70.58 ± 21.26), and the percentage of individuals with adequate well-being followed the same trend (32.6% vs. 15.8%), with *p*-value <0.001 statistically significant in both cases. Table 2 provides detailed information on factors differences among our study population.

For overall digestive problems, total energy and most macronutrients were broadly comparable between groups. However, participants reporting digestive problems showed a slightly lower fat intake (47.81 ± 11.70 vs. 48.73 ± 12.08 g/day; *p* < 0.001), and higher fiber intake (26.41 ± 7.62 vs. 25.84 ± 7.58 g/day; *p* < 0.001). In the food-group analysis, the digestive-problems group reported higher weekly consumption of dairy products (6.08 ± 1.79 vs. 5.91 ± 1.86; *p* < 0.001) and sweet pastries (3.16 ± 2.45 vs. 2.87 ± 2.25; *p* < 0.001), as well as a modestly higher intake of fish (2.53 ± 1.28 vs. 2.43 ± 1.19; *p* < 0.001). Most other food groups (fruit, vegetables, whole grains, legumes, meat, and fast food) did not differ meaningfully, while processed meat intake was slightly lower among those with digestive problems (*p* = 0.032).

Across specific digestive conditions, associations were more heterogeneous and generally smaller in magnitude. Constipation was associated with lower energy, carbohydrate, and fat intake (*p* = 0.026, 0.028, and 0.003, respectively) and lower fruit consumption (*p* = 0.026). Laxative use was linked to higher protein and fiber intake (*p* = 0.013 and 0.002) and to differences in selected food groups (e.g., vegetables/fish and sweet pastries; *p* < 0.01). For stomach medication use, several macronutrients differed significantly (energy, carbohydrates, protein, and fat; *p* = 0.009), alongside higher consumption of dairy products and sweet pastries (*p* < 0.001). In contrast, gastric ulcers showed relatively few consistent associations, with differences appearing mainly in certain food groups (notably dairy products and sweet pastries) rather than in macronutrient intake. Table 3 and Table 4 show total macronutrient intake and the mean weekly food consumption, respectively, across groups.

### 3.4. Bivariate Correlation

When analyzing correlations between continuous variables, significant negative correlations were observed between age and BMI (r = −0.194, *p* < 0.001), emotional well-being (r = −0.171, *p* < 0.001), and fat intake (r = −0.243, *p* < 0.001). Smoking was significantly correlated with alcohol consumption (r = 0.234, *p* < 0.001). Additionally, daily walking minutes were correlated with moderate PA (r = 0.304, *p* < 0.001). Finally, WHO-5 well-being scores and PHQ-8 psychiatric symptom scores showed a strong negative correlation (r = −0.670, *p* < 0.001).

Regarding the weekly food-frequency variables, age showed an overall shift toward a less “processed” pattern: it was inversely correlated with meat (r = −0.150), eggs (r = −0.086), processed meat (r = −0.167), sweet pastries (r = −0.041), and especially fast food (r = −0.421), while being positively correlated with fish (r = 0.145) and dairy products (r = 0.065). In parallel, total energy intake was positively correlated with higher weekly consumption across most food groups, including fruit (r = 0.611), whole grains (r = 0.495), vegetables (r = 0.489), legumes (r = 0.346), dairy products (r = 0.359), sweet pastries (r = 0.271), and fast food (r = 0.273). Finally, fiber intake correlated most strongly with higher weekly consumption of legumes (r = 0.596), fruit (r = 0.453), vegetables (r = 0.360), and whole grains (r = 0.351). Table 5 presents the correlations between continuous variables.

### 3.5. Factors Associated with Digestive Problems

In the multiple logistic regression model, factors such as age, female sex, being a non-smoker or former smoker, higher PHQ-8 scores, protein intake, and sweet pastry intake were associated with the recurrence of digestive problems (OR > 1 with *p*-values <0.05). Conversely, higher well-being scores and fiber intake were associated with lower odds of reporting digestive problems (OR < 1 and *p*-values < 0.001). Table 6 presents simple and multiple logistic regression models for factors associated with digestive problems. An initial multivariable logistic regression model was performed including WHO-5 and PHQ-8 scores. Given the strong inverse correlation between these measures, two additional models were subsequently fit to minimize collinearity: one excluding PHQ-8 score, and a second excluding WHO-5 score. The direction and magnitude of associations for the remaining covariates were consistent across both specifications.

## 4. Discussion

In the Spanish population, digestive problems show variable prevalence, reaching the highest rates in regions such as Murcia and the lowest in the autonomous cities of Ceuta and Melilla. The main factors associated with these problems were older age, female sex, non-smoking status or being a former smoker, depression, higher protein, sweet pastries, and dairy products intake. Conversely, emotional well-being, fiber, and legume consumption were associated with lower odds of digestive problems.

Regarding the prevalence of digestive problems, previous Spanish population-based study [13] reported substantial regional heterogeneity across the country, which is consistent with the findings of the present study. Spain is administratively divided into 17 Autonomous Communities, which differ in demographic structure, lifestyle factors, and health service utilization. In that prior study [13], the highest prevalence was reported in Navarra and Baleares—a Mediterranean island region—and reported lower overall prevalence rates than those observed in EsdE 2023. These discrepancies may be explained by factors including survey year, regional sample sizes, the characteristics of the study population used in that research, survey methods, healthcare access, or unmeasured confounders [13].

Consistent with previous studies [44,46], factors such as advancing age have been associated with the onset of digestive problems, particularly IBD [44] and dyspepsia [46]. This may be explained by multiple mechanisms, including reduced gastrointestinal motility, degeneration of intestinal villi, or the need for medications such as nonsteroidal anti-inflammatory drugs [47]. Regarding sex as a factor associated with digestive disorders, similar to the present analysis, previous publications report female sex as a risk factor for conditions such as IBS, dyspepsia, constipation, and abdominal pain [48,49].

On the other hand, tobacco use as a gastrointestinal risk factor remains controversial [16,17,18,19]. As in the present study, smoking may act as a factor associated with lower odds against digestive problems such as ulcerative colitis [19]; however, tobacco use is associated with higher recurrence of Crohn’s disease [19], gastric ulcer, and constipation [16,17,18]. This discrepancy with the literature may be explained by reverse causality, whereby individuals with digestive problems tend to quit smoking due to medical advice or worsening symptoms. Another explanation is that, in the present study, digestive problems encompass multiple conditions rather than a single pathology.

The presence of emotional problems in relation to various digestive disorders has been widely studied in previous publications [27,29,50]. This association is bidirectional and is primarily mediated by the gut–brain–microbiota axis [30,48]. These findings are consistent with the results of the present analysis, which observed that depression was associated with digestive problems, and was more prevalent among participants with gastric ulcer and among those requiring medication for diarrhea or constipation.

Finally, regarding diet, it plays a highly influential role in gastrointestinal function [33,36,37]. High-protein diets have been associated with changes in gut microbiota—specifically increasing the number of proteolytic species—and increased intestinal permeability, both of which trigger pro-inflammatory processes that compromise digestive health [51,52]. These findings align with the present analysis, as high-protein intake was identified as a risk factor for digestive problems; however, protein source and overall dietary context are likely to be important. Specifically, participants reporting overall digestive problems consumed fish and seafood. This pattern observed in our analysis contrasts with current literature, in which fish and other seafood consumption is generally considered a marker of a healthier dietary profile, and prevents incidence of IBD [53]. Sweet pastry consumption was also more frequent in participants with digestive problems and emerged as a significant risk factor, which is consistent with a western-type dietary pattern characterized by higher intakes of refined carbohydrates, and ultra processed foods [37].

Furthermore, consistent with our findings, higher fiber intake and fiber-derived metabolites have been associated with fewer digestive symptoms and better gastrointestinal function [34,44,54]. This has been reported in digestive conditions such as IBD [34,44,54], IBS [54], and functional disorders such as gastrointestinal transit imbalances [54]. In our food-group analysis, fruit consumption frequency was lower among participants with constipation, and vegetable consumption frequency was higher among those who reported laxative use. In the latter case, as also observed for tobacco use, this pattern may reflect reverse causality and/or behavioral changes after symptom onset, since individuals experiencing constipation may temporarily increase vegetable intake as a short-term self-management strategy or following clinical advice.

### Limitations and Strengths

This study has several limitations. First, digestive disorders were grouped together, so individual conditions were not analyzed separately. Additionally, the cross-sectional design does not allow for establishing causal or temporal relationships. A limitation of the FFQ is that did not explicitly assess the use of seasonings fats such as olive oil used in cooking or dressing foods. Consequently, total energy intake and fat intake—particularly monounsaturated fat—may be underestimated in participants who regularly use olive oil or other added fats. Finally, because covariates were selected using bivariate significance, residual confounding may remain.

On the other hand, the study presents notable strengths. The database used included a nationally representative sample, enabling the calculation of prevalence estimates at the national level. Furthermore, the dataset was designed using a stratified probabilistic sample method which allows the results to be extrapolated to the general population. Finally, validated instruments were employed for data collection, enhancing the reliability of the findings.

## 5. Conclusions

Digestive problems exhibit variable prevalence within the Spanish population, with the highest rates observed in regions such as Murcia and Extremadura. The analysis revealed that factors associated with these conditions include older age, female sex, depression, non-smoking status, and higher intakes of total protein, sweet pastries, and dairy products. Conversely, higher fiber and legume intake, as well as higher well-being scores were associated with reduced odds of gastrointestinal problems.

The findings of this study may help inform approaches to digestive health by highlighting several health behaviors that are associated with digestive problems in our study sample. Understanding these associations is a crucial step toward improving population health. The findings of this analysis may support an integrated health approach aimed at the prevention and management of digestive problems, as they encompass multiple dimensions of health, including nutrition, emotional well-being, harmful habits, and PA.

## Figures and Tables

**Table 1:** Sociodemographic variables.

Variable	Digestive Problems			*p*-Value
	Yes	No	Non-Specified	
*Age, mean ± SD*	66.3 ± 16.69	57.79 ± 18.07	44.17 ± 16.44	<0.001
*Sex, n (%)*				
Male	1437 (37.2)	4172 (43.2)	8293 (40.2)	<0.001
Female	2423 (62.8)	5486 (56.8)	12,337 (59.8)	
*Marital status, n (%)*				
Single	657 (17.1)	2487 (25.9)	7303 (35.4)	<0.001
Married	1848 (48.0)	4721 (49.1)	9248 (45.0)	
Widowed	956 (24.9)	1415 (14.7)	2331 (11.3)	
Separated/Divorced	385 (10.0)	983 (10.2)	1712 (8.3)	
*Educational level, n (%)*				
No formal education	93 (2.4)	87 (0.9)	124 (0.6)	<0.001
Primary education	1127 (29.2)	1972 (19.8)	4518 (21.9)	
Secondary education	1806 (46.8)	5167 (53.5)	8953 (43.4)	
University education	834 (21.6)	2492 (25.8)	7035 (34.1)	
*Employment status, n (%)*				
Student	17 (0.9)	182 (3.7)	5199 (25.2)	<0.001
Employed	643 (32.6)	2322 (47.7)	10,274 (49.8)	
Unemployed	131 (6.6)	409 (8.4)	1630 (7.9)	
Retired/Pensioner	1180 (59.9)	1995 (40.2)	3527 (17.1)	
*Social class, n (%)*				
Class 1	421 (10.9)	1211 (12.5)	2682(13)	0.006
Class 2	238 (6.2)	685 (7.1)	1506 (7.3)	
Class 3	783 (20.3)	1870 (19.4)	3920 (19)	
Class 4	490 (12.7)	1250 (12.9)	2579 (12.5)	
Class 5	1250 (35.7)	2977 (34.3)	6519 (31.6)	
Class 6	527 (14.2)	1278 (13.8)	3424 (16.6)	

**Table 2:** Differences in exposure to health-related factors between groups.

Variable	Digestive Problems		*p*-Value	Gastric Ulcer		*p*-Value	Constipation		*p*-Value	Laxatives Prescription		*p*-Value	Astringent Drug Prescription		*p*-Value	Stomach Medication Prescription		*p*-Value
	Yes	No		Yes	No		Yes	No		Yes	No		Yes	No		Yes	No	
*BMI, mean ± SD*	26.96 ± 4.82	26.32 ± 4.44	<0.001	26.90 ± 4.74	26.30 ± 4.19	0.052	26.38 ± 5.18	26.34 ± 4.50	0.321	26.40 ± 4.95	26.53 ± 4.54	0.037	25.80 ± 4.33	26.53 ± 4.57	0.578	27.55 ± 4.85	26.32 ± 4.46	<0.001
*Weight status, n (%)*																		
Underweight	51 (1.4)	147 (1.6)	<0.001	5 (1.4)	2 (0.6)	0.111	22 (2.2)	3 (2.6)	0.925	13 (2.3)	179 (1.5)	0.230	3 (1.6)	189 (1.5)	0.350	35 (1.3)	157 (1.6)	<0.001
Normal weight	1313 (35.8)	3688 (40.2)		127 (35.5)	130 (39.9)		425 (43.2)	46 (40)		233 (40.5)	4680 (38.7)		84 (44.2)	4829 (38.7)		923 (33.3)	3985 (40.2)	
Overweight	1485 (40.4)	3698 (40.3)		148 (41.3)	144 (44.2)		339 (34.5)	42 (36.5)		214 (37.2)	4904 (40.5)		74 (38.9)	5042 (40.4)		1139 (41.1)	3981 (40.2)	
Obesity	824 (22.4)	1651 (17.9)		78 (21.8)	50 (15.3)		197 (20.0)	24 (20.9)		115 (20.0)	2342 (19.3)		29 (15.3)	2430 (19.5)		674 (24.3)	1780 (18.0)	
*Smoking status, n (%)*																		
Yes	544 (14.1)	1882 (19.5)	<0.001	55 (15.1)	74 (22.2)	0.006	135 (13.0)	20 (16.4)	0.558	75 (12.2)	2311 (18.2)	<0.001	30 (15.5)	2355 (18.0)	0.209	386 (13.3)	1996 (19.2)	<0.001
No	2071 (53.8)	5288 (54.9)		196 (52.9)	139 (41.7)		629 (60.4)	72 (59.0)		359 (58.5)	6898 (54.4)		100 (51.5)	7156 (54.6)		1544 (53.3)	5710 (54.9)	
Former smoker	1233 (32.0)	2467 (25.6)		117 (32.1)	120 (36.0)		277 (26.6)	30 (24.6)		180 (29.3)	3481 (27.4)		64 (33.0)	3597 (27.4)		968 (33.4)	2690 (25.9)	
*Cigarettes per day, mean ± SD*	13.77 ± 13.55	14.06 ± 13.44	0.977	13.89 ± 7.86	15.73 ± 14.68	0.357	10.17 ± 6.18	13.74 ± 13.42	0.329	15.51 ± 17.12	13.68 ± 13.08	0.118	13.69 ± 13.14	17.48 ± 18.59	0.019	13.30 ± 10.29	13.84 ± 13.74	0.189
*Alcoholic drinks per week, mean ± SD*	7.87 ± 6.77	7.96 ± 7.34	0.716	9.08 ± 6.86	8.62 ± 7.45	0.945	6.55 ± 5.24	6.73 ± 5.65	0.179	7.23 ± 6.54	7.99 ± 7.24	0.175	9.25 ± 11.61	7.94 ± 7.13	0.003	8.05 ± 7.01	7.93 ± 7.26	0.947
*PA (IPAQ), n (%)*																		
Low	2656 (68.8)	5719 (59.2)	<0.001	249 (68.0)	215 (64.2)	0.526	700 (67.1)	82 (66.7)	0.874	418 (67.9)	7863 (61.8)	0.007	118 (60.5)	8158 (62.1)	0.901	2075 (71.4)	6194 (59.4)	<0.001
Moderate	572 (14.8)	1914 (19.8)		50 (13.7)	54 (16.1)		190 (18.2)	21 (17.1)		102 (16.6)	2334 (18.4)		37 (19.0)	2401 (18.3)		374 (12.9)	2063 (19.8)	
Vigorous	632 (16.4)	2025 (21.0)		67 (18.3)	66 (19.7)		153 (14.7)	20 (16.3)		96 (15.6)	2521 (19.8)		40 (20.5)	2577 (19.6)		457 (15.7)	2162 (20.8)	
*Minutes of PA per day, mean ± SD*																		
Walking	45.88 ± 34.44	48.69 ± 35.74	<0.001	50.13 ± 36.42	49.34 ± 36.44	0.973	45.64 ± 33.78	46.91 ± 38.11	0.103	44.44 ± 34.22	48.02 ± 35.37	0.188	46.66 ± 34.72	47.88 ± 35.34	0.924	44.82 ± 33.54	48.61 ± 35.72	<0.001
Moderate PA	51.49 ± 41.94	57.56 ± 44.35	0.470	60.83 ± 45.43	62.00 ± 47.76	0.766	48.89 ± 35.14	47.50 ± 42.31	0.876	41.46 ± 32.91	56.85 ± 44.18	0.030	72.50 ± 52.15	56.21 ± 43.84	0.205	54.58 ± 44.94	56.71 ± 43.81	0.688
Vigorous PA	31.84 ± 83.47	37.32 ± 74.36	<0.001	31.79 ± 67.11	34.09 ± 88.06	0.631	33.69 ± 95.06	37.18 ± 98.43	0.804	31.69 ± 75.00	35.61 ± 75.08	0.574	41.66 ± 90.80	35.37 ± 74.87	0.083	28.66 ± 77.59	37.40 ± 74.35	<0.001
*PHQ-8 score, mean ± SD*	7.10 ± 5.98	4.70 ± 4.61	<0.001	7.48 ± 5.92	5.91 ± 5.22	0.019	8.38 ± 6.28	6.96 ± 5.55	0.002	8.73 ± 6.43	5.22 ± 5.030	<0.001	8.28 ± 6.66	5.34 ± 5.12	<0.001	7.07 ± 5.98	4.90 ± 4.79	<0.001
*Depression, n (%)*																		
None	2628 (72.1)	8043 (86.1)	<0.001	236 (68.0)	249 (78.8)	0.007	623 (63.8)	85 (72.6)	0.148	353 (62.1)	10,178 (83.1)	<0.001	115 (64.2)	10,413 (82.4)	<0.001	1994 (72.3)	8532 (84.9)	<0.001
Depression	816 (22.4)	1150 (12.3)		95 (27.4)	59 (18.7)		283 (29.0)	27 (23.1)		163 (28.7)	1771 (14.5)		49 (27.4)	1888 (14.9)		608 (15.6)	(66.6)	
Severe depression	203 (5.6)	149 (1.6)		16 (4.6)	8 (2.5)		70 (7.2)	5 (4.3)		52 (9.2)	298 (2.4)		15 (8.4)	335 (2.7)		156 (5.7)	192 (1.9)	
*WHO-5 score, mean ± SD*	59.47 ± 25.11	70.58 ± 21.26	<0.001	59.22 ± 25.10	63.15 ± 22.61	0.036	54.25 ± 26.37	59.97 ± 23.22	0.015	51.58 ± 26.00	68.17 ± 22.55	<0.001	53.64 ± 28.24	67.62 ± 22.82	<0.001	59.62 ± 24.86	69.61 ± 21.92	<0.001
*Mental well-being level, n (%)*																		
Low well-being	1260 (32.6)	1529 (15.8)	<0.001	119 (32.5)	83 (24.8)	0.024	440 (42.2)	35 (28.5)	0.003	281 (45.6)	2469 (19.4)	<0.001	86 (44.1)	2661 (20.3)	<0.001	937 (32.2)	1806 (17.3)	<0.001
Adequate well-being	2600 (67.4)	8129 (84.2)		247 (67.5)	252 (75.2)		603 (57.8)	88 (71.5)		335 (54.4)	10,249 (80.6)		109 (55.9)	10,475 (79.7)		1969 (67.8)	8613 (82.7)	

**Table 3:** Macronutrient intake and digestive problems.

Variable	Digestive Problems		*p*-Value	Cohen’s d [95% CI]	Gastric Ulcer		*p*-Value	Cohen’s d [95% CI]	Constipation		*p*-Value	Cohen’s d [95% CI]	Laxatives Prescription		*p*-Value	Cohen’s d [95% CI]	Astringent Drug Prescription		*p*-Value	Cohen’s d [95% CI]	Stomach Medication Prescription		*p*-Value	Cohen’s d [95% CI]
	Yes	No			Yes	No			Yes	No			Yes	No			Yes	No			Yes	No		
Daily kcal	1590.95 ± 379.82	1591.77 ± 397.06	0.913	0.002 [−0.035, 0.040]	1601.46 ± 399.71	1613.58 ± 413.18	0.420	0.030 [−0.119, 0.178]	1594.60 ± 392.02	1610.91 ± 565.26	0.026	0.039 [−0.148, 0.227]	1597.04 ± 423.25	1590.07 ± 388.53	0.061	−0.018 [−0.100, 0.064]	1580.68 ± 392.57	1590.53 ± 390.09	0.068	0.025 [−0.117, 0.168]	1588.80 ± 351.78	1591.29 ± 400.33	0.004	0.006 [−0.035, 0.048]
Carbohydrates (g/day)	226.77 ± 54.16	226.82 ± 56.56	0.958	0.001 [−0.037, 0.039]	228.20 ± 56.95	229.93 ± 58.87	0.420	0.030 [−0.117, 0.180]	227.45 ± 56.00	229.55 ± 80.55	0.028	0.036 [−0.152, 0.223]	227.57 ± 60.31	226.60 ± 55.36	0.062	−0.016 [−0.098, 0.065]	225.24 ± 55.94	226.66 ± 55.58	0.065	0.026 [−0.117, 0.165]	226.40 ± 50.13	226.77 ± 57.04	0.006	0.007 [−0.035, 0.048]
Protein (g/day)	79.49 ± 18.54	79.43 ± 18.62	0.872	−0.003 [−0.041, 0.034]	79.99 ± 20.11	80.29 ± 17.54	0.661	0.016 [−0.113, 0.164]	79.57 ± 18.59	79.51 ± 21.78	0.077	−0.003 [−0.191, 0.185]	79.95 ± 21.34	79.36 ± 18.33	0.013	−0.032 [−0.113, 0.050]	78.93 ± 19.88	79.40 ± 18.45	0.027	0.025 [−0.120, 0.168]	79.40 ± 17.31	79.41 ± 18.79	0.009	0.000 [−0.041, 0.042]
Fat (g/day)	47.81 ± 11.70	48.73 ± 12.08	<0.001	0.077 [0.039, 0.114]	47.48 ± 12.43	48.91 ± 12.49	0.565	0.115 [−0.034, 0.264]	47.49 ± 11.74	48.54 ± 16.07	0.003	0.085 [−0.103, 0.273]	47.36 ± 11.89	48.48 ± 11.93	0.748	0.094 [0.013, 0.176]	48.57 ± 13.34	48.42 ± 11.91	0.008	−0.013 [−0.115, 0.130]	47.80 ± 11.23	48.61 ± 12.11	<0.001	0.068 [0.026, 0.109]
Fiber (g/day)	26.41 ± 7.62	25.84 ± 7.58	<0.001	−0.076 [−0.113, −0.038]	26.52 ± 7.74	26.62 ± 7.68	0.675	0.013 [−0.136, 0.162]	26.61 ± 8.00	25.79 ± 9.31	0.263	−0.100 [−0.287, 0.088]	26.79 ± 8.34	25.95 ± 7.53	0.002	−0.111 [−0.192, −0.029]	25.65 ± 8.46	25.99 ± 7.56	0.106	0.045 [−0.097, 0.188]	26.40 ± 7.35	25.88 ± 7.63	0.047	−0.068 [−0.110, −0.027]

Data presented as means ± SD. CI, Confidence Interval.

**Table 4:** Weekly food frequency intake and digestive problems.

Food Group	Digestive Problems		*p*-Value	Cohen’s d [95% CI]	Gastric Ulcer		*p*-Value	Cohen’s d [95% CI]	Constipation		*p*-Value	Cohen’s d [95% CI]	Laxatives Prescription		*p*-Value	Cohen’s d [95% CI]	Astringent Drug Prescription		*p*-Value	Cohen’s d [95% CI]	Stomach Medication Prescription		*p*-Value	Cohen’s d [95% CI]
	Yes	No			Yes	No			Yes	No			Yes	No			Yes	No			Yes	No		
Fruit	13.83 ± 4.53	12.83 ± 4.86	0.288	−0.030 [−0.067, 0.008]	14.91 ± 4.56	13.88 ± 4.50	0.757	−0.027 [−0.176, 0.121]	13.73 ± 4.98	16.91 ± 6.32	0.026	0.107 [−0.080, 0.293]	14.41 ± 5.36	13.02 ± 4.21	0.896	−0.042 [−0.123, 0.040]	11.07 ± 8.71	13.11 ± 8.99	0.459	0.061 [−0.081, 0.203]	13.54 ± 8.56	12.96 ± 7.99	0.134	−0.017 [−0.058, 0.024]
Meat	3.55 ± 1.68	3.63 ± 1.67	0.550	0.047 [0.009, 0.084]	3.43 ± 1.73	3.75 ± 1.74	0.082	0.184 [0.035, 0.333]	3.54 ± 1.78	3.60 ± 1.87	0.296	0.038 [−0.149, 0.225]	3.48 ± 1.75	3.61 ± 1.66	0.177	0.079 [−0.001, 0.160]	3.86 ± 1.95	3.60 ± 1.66	<0.001	−0.154 [−0.296, −0.012]	3.54 ± 1.64	3.62 ± 1.68	0.002	0.051 [0.010, 0.092]
Egg	79.49 ± 18.54	79.43 ± 18.62	0.631	0.010 [−0.027, 0.048]	2.99 ± 1.38	3.21 ± 1.67	<0.001	0.145 [−0.004, 0.293]	3.16 ± 1.53	3.07 ± 1.63	0.488	−0.061 [−0.248, 0.126]	3.13 ± 1.49	3.05 ± 1.43	0.018	−0.054 [−0.135, 0.027]	3.35 ± 1.74	3.86 ± 1.95	<0.001	−0.209 [−0.350, −0.067]	3.01 ± 1.40	3.07 ± 1.44	0.016	0.046 [0.005, 0.087]
Fish/seafood	2.53 ± 1.28	2.43 ± 1.19	<0.001	−0.081 [−0.118, −0.043]	2.42 ± 1.22	2.58 ± 1.27	0.275	0.130 [−0.019, 0.278]	2.54 ± 1.37	2.54 ± 1.45	0.270	0.001 [−0.185, 0.188]	2.62 ± 1.40	2.45 ± 1.20	<0.001	−0.141 [−0.222, −0.060]	2.51 ± 1.41	2.45 ± 1.21	0.002	−0.048 [−0.190, 0.094]	2.53 ± 1.25	2.43 ± 1.19	<0.001	−0.075 [−0.117, −0.034]
Whole grains	9.15 ± 2.61	9.09 ± 2.64	0.142	−0.023 [−0.060, 0.015]	9.36 ± 2.54	9.12 ± 2.78	0.224	−0.087 [−0.236, 0.061]	8.97 ± 2.86	9.22 ± 2.65	0.567	0.089 [−0.098, 0.276]	8.95 ± 2.79	9.11 ± 2.62	0.035	0.060 [−0.021, 0.140]	8.95 ± 2.73	9.11 ± 2.63	0.804	0.060 [−0.082, 0.201]	9.21 ± 2.53	9.08 ± 2.66	<0.001	−0.049 [−0.091, −0.008]
Vegetables	8.19 ± 3.65	8.18 ± 3.54	0.679	0.000 [−0.038, 0.037]	8.36 ± 3.73	6.68 ± 2.90	0.136	−0.063 [−0.212, 0.085]	8.39 ± 5.89	7.08 ± 5.63	0.472	−0.045 [−0.232, 0.142]	10.47 ± 6.93	7.98 ± 6.03	0.007	−0.078 [−0.159, 0.003]	7.00 ± 6.93	8.17 ± 7.09	0.467	0.036 [−0.106, 0.178]	7.63 ± 6.52	8.30 ± 6.64	0.055	0.021 [−0.020, 0.062]
Legumes	2.51 ± 1.22	2.55 ± 1.19	0.332	0.037 [0.000, 0.075]	2.52 ± 1.26	2.64 ± 1.22	0.599	0.093 [−0.055, 0.242]	2.47 ± 1.22	2.38 ± 1.42	0.032	−0.072 [−0.259, 0.114]	2.49 ± 1.28	2.54 ± 1.19	0.131	0.038 [−0.043, 0.119]	2.42 ± 1.42	2.54 ± 1.19	0.026	0.099 [−0.043, 0.241]	2.52 ± 1.21	2.54 ± 1.91	0.534	0.019 [−0.022, 0.061]
Processed meat	2.44 ± 1.77	2.62 ± 1.80	0.032	0.100 [0.062, 0.137]	2.54 ± 1.90	2.47 ± 1.79	0.247	−0.040 [−0.189, 0.108]	2.31 ± 1.81	2.53 ± 2.02	0.137	0.123 [−0.064, 0.310]	2.37 ± 1.80	2.57 ± 1.79	0.845	0.113 [0.032, 0.194]	2.53 ± 1.93	2.56 ± 1.79	0.071	0.019 [−0.122, 0.161]	2.44 ± 1.72	2.59 ± 1.80	<0.001	0.086 [0.044, 0.127]
Dairy products	6.08 ± 1.79	5.91 ± 1.86	<0.001	−0.095 [−0.132, −0.057]	5.95 ± 1.99	6.21 ± 1.66	<0.001	0.142 [−0.007, 0.291]	6.00 ± 1.84	6.04 ± 1.81	0.746	0.025 [−0.161, 0.212]	6.04 ± 1.76	5.95 ± 1.84	0.074	−0.047 [−0.128, 0.033]	5.69 ± 2.19	5.96 ± 1.83	<0.001	0.146 [0.005, 0.288]	6.17 ± 1.72	5.90 ± 1.87	<0.001	−0.145 [−0.187, −0.104]
Sweet pastries	3.16 ± 2.45	2.87 ± 2.25	<0.001	−0.125 [−0.163, −0.088]	2.92 ± 2.39	3.21 ± 2.53	0.020	0.118 [−0.031, 0.226]	3.21 ± 2.50	3.08 ± 2.47	0.338	−0.055 [−0.241, 0.132]	3.22 ± 2.46	2.94 ± 2.30	<0.001	−0.120 [−0.201, −0.039]	3.36 ± 2.56	2.95 ± 2.30	<0.001	−0.180 [−0.322, −0.038]	3.22 ± 2.45	2.88 ± 2.26	<0.001	−0.146 [−0.186, −0.104]
Fast food	0.86 ± 1.08	1.11 ± 1.09	0.411	0.230 [0.192, 0.267]	0.92 ± 1.20	0.84 ± 1.05	0.051	−0.065 [−0.213, 0.084]	0.82 ± 1.10	0.90 ± 1.22	0.798	0.072 [−0.116, 0.259]	0.78 ± 1.04	1.04 ± 1.09	0.964	0.242 [0.161, 0.323]	0.97 ± 1.30	1.03 ± 1.09	0.019	0.057 [−0.085, 0.199]	0.83 ± 1.07	1.09 ± 1.09	0.671	0.235 [0.194, 0.277]

Data presented as means ± SD. CI, Confidence Interval.

**Table 5:** Correlations between continuous variables.

	Age	BMI	Cigarettes/Day	Alcoholic Drinks/Week	PHQ-8 Score	WHO-5 Score	Daily Walking Minutes	Daily Moderate PA Minutes	Daily Vigorous PA Minutes	Daily Kcal Intake	Daily Carbohydrate Intake (g)	Daily Protein Intake (g)	Daily Fat Intake (g)	Daily Fiber Intake (g)	Weekly Fruit Intake	Weekly Meat Intake	Weekly Eggs Intake	Weekly Fish/Seafood Intake	Weekly Whole Grains Intake	Weekly Vegetable Intake	Weekly Legume Intake	Weekly Processed Meat intake	Weekly Dairy Product Intake	Weekly Sweet Pastries Intake	Weekly Fast-Food Intake
Age	-	−0.194 **	−0.101 **	0.105 **	0.146 **	−0.171 **	−0.008	0.049 *	−0.171 *	−0.101 **	−0.101 **	−0.104 **	−0.243 **	0.091 **	0.047 **	−0.150 **	−0.086 **	0.145 **	−0.037 **	0.014 *	0.046 **	−0.167 **	0.065 **	−0.041 **	−0.421 **
BMI		-	0.012	0.083 **	0.076 **	−0.065 **	−0.045 **	−0.019	−0.090 **	0.021 **	0.021 **	0.020 **	0.015 *	−0.010	−0.011	0.024 **	−0.040 **	−0.008	0.000	−0.012	0.006	0.033 **	0.010	−0.014 *	−0.026 **
Cigarettes/day			-	0.234 **	0.008	−0.011	−0.027 **	−0.011	−0.045 **	0.000	0.000	0.002	−0.076 **	−0.098 **	−0.048 **	0.029 **	−0.018 *	−0.070 **	0.026 **	−0.023 **	−0.036 **	0.095 **	−0.002	0.035 **	0.140 **
Alcoholic drinks/week				-	−0.001	0.007	0.025	0.001	−0.063 **	−0.042 **	−0.042 **	−0.042 **	0.002	−0.098 **	−0.047 **	−0.017	−0.008	−0.025 *	0.049 **	−0.036 **	−0.027 *	0.106 **	−0.034 **	0.000	0.006
PHQ-8 score					-	−0.670 **	−0.042 **	−0.012	−0.087 **	−0.069 **	−0.069 **	−0.076 **	−0.073 **	−0.028 **	0.001	−0.069 **	−0.020 **	−0.018 *	−0.115 **	0.005	0.038 **	−0.062 **	−0.098 **	0.019 **	−0.014 *
WHO-5 score						-	0.095 **	0.052 *	0.040 **	0.082 **	0.082 **	0.091 **	0.083 **	0.040 **	−0.012	0.023 **	0.018 **	0.040 **	0.113 **	−0.013	−0.008	0.047 **	0.055 **	−0.021 **	0.083 **
Daily walking minutes							-	0.304 **	0.050 **	0.021 **	0.022 **	0.022 **	−0.018 **	−0.074 **	0.040 **	−0.037 **	0.016 *	0.051 **	−0.023 **	0.017 *	0.029 **	−0.020 **	−0.007	−0.026 **	−0.026 **
Daily moderate PA minutes								-	0.118 **	−0.009	−0.010	−0.013	−0.015	0.016	−0.005	−0.045	−0.034	−0.035	−0.007	−0.015	0.026	0.033	−0.018	−0.059 *	0.026
Daily vigorous PA minutes									-	0.050 **	0.050 **	0.053 **	0.061 **	0.048 **	0.028 **	0.062 **	0.059 **	0.020 **	−0.020 **	0.024 **	0.029 **	0.046 **	−0.017 *	−0.015 *	0.054 **
Daily kcal intake										-	1.000 **	0.978 **	0.856 **	0.786 **	0.611 **	0.353 **	0.353 **	0.270 **	0.495 **	0.489 **	0.346 **	0.291 **	0.359 **	0.271 **	0.273 **
Daily carbohydrate intake (g)											-	0.978 **	0.856 **	0.785 **	0.611 **	0.353 **	0.353 **	0.270 **	0.495 **	0.489 **	0.346 **	0.291 **	0.359 **	0.271 **	0.273 **
Daily protein intake (g)												-	0.856 **	0.786 **	0.494 **	0.377 **	0.376 **	0.286 **	0.529 **	0.520 **	0.368 **	0.311 **	0.387 **	0.289 **	0.291 **
Daily fat intake (g)													-	0.516 **	0.357 **	0.448 **	0.461 **	0.189 **	0.388 **	0.306 **	0.232 **	0.514 **	0.437 **	0.413 **	0.515 **
Daily fiber intake (g)														-	0.453 **	0.179 **	0.274 **	0.332 **	0.351 **	0.360 **	0.596 **	0.117 **	0.240 **	0.129 **	0.026 **
Weekly fruit intake															-	0.027 **	0.061 **	0.110 **	0.043 **	0.489 **	0.090 **	0.000	0.037 **	0.002	−0.036 **
Weekly meat intake																-	0.321 **	0.106 **	0.208 **	0.049 **	0.068 **	0.182 **	0.034 **	0.079 **	0.102 **
Weekly eggs intake																	-	0.210 **	0.141 **	0.077 **	0.193 **	0.103 **	0.023 **	0.058 **	0.067 **
Weekly fish/seafood intake																		-	0.039 **	0.103 **	0.222 **	0.019 **	0.033 **	0.018 *	−0.035 **
Weekly whole grains intake																			-	0.051 **	0.073 **	0.193 **	0.223 **	0.127 **	0.076 **
Weekly vegetable intake																				-	0.112 **	0.018 **	0.020 **	−0.010	−0.006
Weekly legume intake																					-	0.046 **	−0.012	0.043 **	0.026 **
Weekly processed meat intake																						-	0.116 **	0.182 **	0.262 **
Weekly dairy product intake																							-	0.186 **	−0.029 **
Weekly sweet pastries intake																								-	−0.210 **
Weekly fast-food intake																									-

* *p*-value < 0.05, ** *p*-value < 0.001. BMI: Body Mass Index; PA: Physical Activity; PHQ-8: Personal Health Questionnaire Depression Scale; WHO-5: World Health Organization Well Being Index.

**Table 6:** Logistic regression analysis of factors associated with digestive problems.

Variable	Simple Logistic Regression		Multivariable Logistic Regression *		Adjusted OR Excluding PHQ8 Score [95% CI]	*p*-Value	Adjusted OR Excluding WHO5 Score [95% CI]	*p*-Value
	Crude OR [95% CI]	*p*-Value	Adjusted OR [95% CI]	*p*-Value				
*Age*	1.028 [1.025, 1.030]	<0.001	1.026 [1.023, 1.029]	<0.001	1.026 [1.024, 1.029]	<0.001	1.025 [1.022, 1.028]	<0.001
*Sex*								
Male	Reference		Reference		Reference		Reference	
Female	1.155 [1.061, 1.258]	<0.001	1.168 [1.070, 1.276]	<0.001	1.185 [1.088, 1.291]	<0.001	1.148 [1.055, 1.249]	<0.001
*Smoking status*								
Yes	Reference		Reference		Reference		Reference	
No	1.355 [1.216, 1.510]	<0.001	1.240 [1.005, 1.531]	0.045	1.004 [0.891, 1.130]	0.954	1.009 [0.857, 1.095]	0.594
Former smoker	1.729 [1.538, 1.944]	<0.001	1.447 [1.272, 1.647]	<0.001	1.439 [1.269, 1.632]	<0.001	1.444 [1.270, 1.642]	<0.001
*WHO5 score*	0.984 [0.982, 0.987]	<0.001	0.985 [0.982, 0.987]	<0.001	0.979 [0.978, 0.981]	<0.001	Excluded	
*PHQ8 score*	1.036 [1.026, 1.047]	<0.001	1.040 [1.029, 1.051]	<0.001	Excluded		1.087 [1.079, 1.096]	<0.001
*Protein intake*	1.007 [1.003, 1.011]	<0.001	1.016 [1.009, 1.023]	<0.001	Excluded		Excluded	
*Fiber intake*	0.997 [0.988, 1.006]	0.504	0.968 [0.949, 0.987]	<0.001	0.989 [0.815, 0.998]	<0.001	0.991 [0.986, 0.996]	<0.001
*Sweet pastries intake*	1.055 [1.038–1.072]	<0.001	1.058 [1.039–1.077]	<0.001	1.060 [1.042, 1.079]	<0.001	1.059 [1.040, 1.078]	<0.001
*Legume intake*	0.969 [0.939, 1.000]	0.050	0.894 [0.856, 0.933]	<0.001	0.887 [0.850, 0.926]	<0.001	0.895 [0.857, 0.934]	<0.001
*Dairy products intake*	1.055 [1.033, 1.077]	<0.001	1.027 [1.002, 1.053]	0.032	Excluded		1.029 [1.004, 1.054]	<0.001
*Fruit intake*	1.001 [0.998, 1.003]	0.125	Excluded		0.998 [0.996, 1.000]	0.016	Excluded	

CI: Confidence Interval; OR: Odds Ratio; * Model performance: Nagelkerke’s R^2^ value = 0.12.

## Data Availability

The original contributions presented in this study are included in the article. Further inquiries can be directed to the corresponding author.

## Abbreviations

The following abbreviations are used in this manuscript:

IBD	Inflammatory Bowel Disease
IBS	Irritable Bowel Syndrome
PA	Physical Activity
EsDE 2023	2023 Spanish Health Survey
BMI	Body Mass Index
IPAQ	International Physical Activity Questionnaire
PHQ-8	Personal Health Questionnaire Depression Scale
WHO-5	World Health Organization Well-Being Index
FFQ	Food Frequency Questionnaire
BEDCA	Spanish Food Composition Database
SD	Standard Deviation
CI	Confidence Interval
